# Iodine Content and Distribution in Thyroid Specimens from Two Patients with Graves' Disease Pretreated with Either Propylthiouracil or Stable Iodine: Analysis Using X-Ray Fluorescence and Time-of-Flight Secondary Ion Mass Spectrometry

**DOI:** 10.1155/2012/842357

**Published:** 2012-02-09

**Authors:** Marie Hansson, Helena Filipsson Nyström, Svante Jansson, Jukka Lausmaa, Gertrud Berg

**Affiliations:** ^1^Department of Radiation Physics at University of Gothenburg, Sahlgrenska University Hospital, 413 45 Göteborg, Sweden; ^2^Department of Endocrinology, Gröna stråket 8, Sahlgrenska University Hospital, 413 45 Göteborg, Sweden; ^3^Department of Surgery, Sahlgrenska University Hospital, 413 45 Göteborg, Sweden; ^4^Chemistry and Materials Technology, SP Technical Research Institute of Sweden, P.O.Box 857, 501 15 Boras, Sweden; ^5^Department of Oncology, Institute of Clinical Sciences Sahlgrenska Academy at University of Gothenburg, Sahlgrenska University Hospital, 413 45 Göteborg, Sweden

## Abstract

Patients with Graves' disease can be medically prepared before surgery in different ways, which may have various effects on iodine stores. Thyroid specimens were collected at surgery from two patients pretreated with propylthiouracil (PTU) and stable iodine, respectively. A quantitative analysis of iodine content was performed using X-ray fluorescence (XRF) in frozen tissue and a qualitative analysis of aldehyde-fixed material with Time-of-Flight Secondary Ion Mass Spectrometry (TOF-SIMS). Iodine concentrations were 0.9 mg/mL and 0.5 mg/mL in the thyroid tissue from the patients treated with PTU and stable iodine respectively. TOF-SIMS showed iodine in the follicle lumina in both. However, in the PTU case, iodine was also seen within the thyrocytes indicating accumulation of iodinated compounds from uninhibited hormone release. XRF and TOF-SIMS can be used to follow iodine distribution within the thyroid and the intricate processes following the different medical treatment alternatives in Graves' disease.

## 1. Introduction

 The thyroid iodine content is of major importance in different thyroid diseases and is likely to affect their treatment. The individual size of the thyroid iodine pool is affected by many factors including diet, gender, and age [1] but may also be affected by hyperthyroidism, such as Graves' disease and the treatment of the hyperthyroid state. Before surgery of Graves' disease, the patient is rendered euthyroid either by giving antithyroid drugs (ATDs) or by administering stable iodine. ATDs are used in most cases but stable iodine is preferred when a rapid response on hormonal release is requested. ATDs affect hormonal production by interfering with the enzyme thyroid peroxidase (TPO) at the apical membrane of the thyrocytes, thus blocking the iodination of thyroglobulin (Tg) [2]. Stable iodine inhibits endocytosis of hormone containing Tg (from the follicle lumina into the thyrocytes), the so-called Plummer effect [3, 4], and also affects hormone production at the apical membrane, the Wolff-Chaikoff effect [5].

 The different medical pretreatments may have implications on the thyroid iodine uptake and the thyroid iodine pool. The stored iodine concentration in the thyroid can be studied with quantitative X-ray fluorescence analysis (XRF), and knowledge about iodine distribution can be acquired with time of flight secondary ion mass spectrometry (TOF-SIMS), which is a method where accumulation of iodine can be determined histologically at a cellular level [6–8]. The aim of the present paper was to study if these methods could be of use to determine the magnitude and location of iodine at the cellular level in Graves' disease in order to add to the understanding of the mechanisms behind medical treatment. 

## 2. Case Presentations

 Thyroid tissue specimens were collected at surgery from two patients with Graves' disease. Specimen 1 was from a 30-year-old woman who was diagnosed with Graves' disease during pregnancy in December 2006. At week twelve of pregnancy, biochemical analyses showed free thyroxine (fT4) 36 pmol/L (normal range 12–22 pmol/L), triiodothyronine (T3) 4.2 nmol/L (normal range 1.3–3.1 nmol/L), serum thyroid stimulating hormone (TSH) 0.007 mU/L (normal range 0.3–4.2 mU/L), and TSH receptor antibodies (TRAb) 9.5 IU/L (normal <1 IU/L). The patient started treatment with 150 mg propylthiouracil (PTU) per day and after four weeks the dose was reduced to 100 mg per day. The patient delivered a healthy baby in June 2007. One month postpartum, fT4 was 30 pmol/L and the PTU dose was increased to 200 mg per day and in October, when the patient had an fT4 of 71 pmol/L, the PTU dose was further increased to 400 mg per day. It was, however, considered that the compliance to the medical treatment was problematic and it was, therefore, decided to refer the patient for surgery. In January 2008, when her biochemical parameters were fT4 25 pmol/L, T3 3.7 nmol/L, TSH 0.005 mU/L, and TRAb 10 IU/L, the patient underwent total thyroidectomy.

 Specimen 2 was from a 25-year-old woman who was diagnosed with Graves' disease in November 2007 after having symptoms of thyrotoxicosis for about 5 months. The diagnosis was confirmed biochemically with fT4 > 100 pmol/L, T3 > 10 nmol/L, TSH 0.005 mU/L, and TRAb 14 IU/L. Initially treatment with thiamazole was planned but after 7.5 weeks of treatment with a combination of thiamazole 30 mg per day and thyroxine the patient developed neutropenia with neutrophiles lower than 0.9 × 10^9^/L. Thus the medical treatment was discontinued, and the patient was referred for thyroidectomy, which was performed three weeks later. Ten days before surgery, the patient received treatment with Lugols solution (I-KI 5% 11 mg iodine three times per day); at surgery, neutrophiles had recovered and biochemical parameters were fT4 23 pmol/L, T3 3.1 nmol/L, and TSH 0.008 mU/L. Total thyroidectomy was performed without complications.

### 2.1. Analysis

 Approximately 4 × 4 mm fresh biopsies from each of the surgically removed thyroid glands were collected immediately and analyzed with XRF or fixated for later analysis with TOF-SIMS. The basic principle of the XRF technique is that stable iodine atoms (^127^I) emit characteristic X-rays when exposed to ionizing radiation from a radioactive source [9]. In the present study, an 11.1 GBq ^241^Am source was used for irradiation. The characteristic X-rays were detected and analyzed with a planar HPGe detector, and the amount of iodine in the sample was calculated from the intensity of the characteristic X-rays compared to measurements from standards as previously reported [9].

 For TOF-SIMS analysis, the tissue samples were histologically prepared by fixation in modified Karnovsky fixative (2% paraformaldehyde, 2.5% glutaraldehyde, and 0.05 M sodium cacodylate buffer pH 7.2). After being stored at 4°C for 1–6 days, the samples were rinsed and postfixed before being dehydrated in a series of ethanol solutions and embedded in Agar 100 resin. Sections were cut (Reichert Ultracut E) for TOF-SIMS analysis (2 *μ*m) and for histological control with light microscopy (1 *μ*m). The light microscopy sections were stained according to Richardson et al. [10].

TOF-SIMS analysis is based on high-resolution mass spectrometric analysis of ions emitted from the sample surface during irradiation with an energetic ion beam. By using focused beams, the technique can be used in a microscopy mode. The negative spectra showed strong signals from phosphate, PO_3_^−^, which have been attributed to phospholipids in cell membranes in previous TOF-SIMS studies [11]. Thus, these signals can be used as an indicator of cell membranes. Overlays of the iodine and PO_3_^−^ signals were used for illustrating the lateral distribution of iodine in relation to cells. All TOF-SIMS analyses were done with a TOF-SIMS IV instrument (IONTOF GmbH, Münster, Germany) using 25 keV Bi_3_^+^ primary ions at an average beam current of 0.2 pA. Emitted positive or negative secondary ions were analyzed and the result was presented as images in a similar way as previously described [8].

### 2.2. Ethics

 Iodine determinations on human tissue were approved by the Ethical Committee of Göteborgs' University and the patients had given their informed consent. The study was performed according to the Declaration of Helsinki.

### 2.3. Results of XRF Measurements

 The iodine concentrations measured with XRF were 0.9 mg/mL and 0.5 mg/mL in tissue from specimen 1 (pretreated with PTU) and 2 (stable iodine), respectively.

### 2.4. Results of TOF-SIMS Analysis

**Figure 1:**
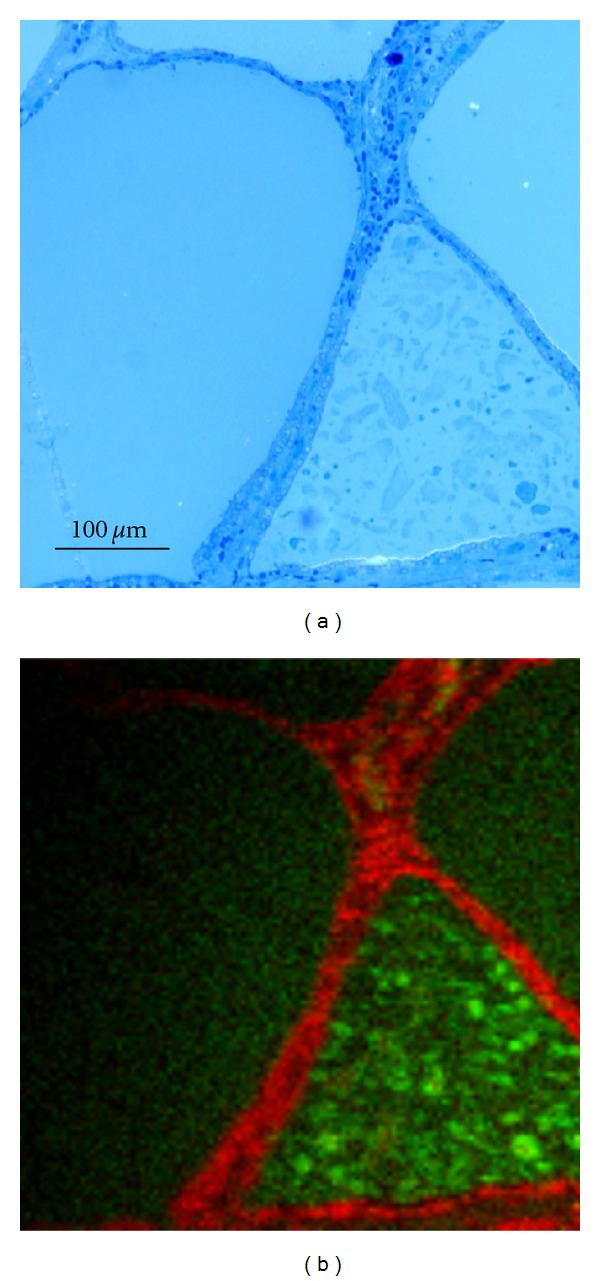
Light microscopy image (a) and TOF-SIMS image (b) of the tissue section from the PTU pretreated patient. The green in the TOF-SIMS image represents the signal from iodine and the red marks PO_3_^−^ (signal from cell membranes). Iodine signals were mainly found within the follicle lumina but also in some areas outside the follicles, presumably in the thyrocytes. The iodine concentration was inhomogeneously distributed within some follicle lumina.

**Figure 2:**
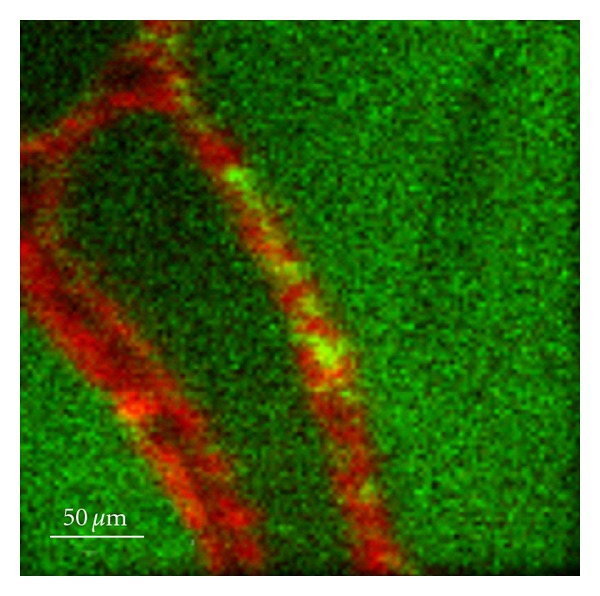
TOF-SIMS image of a second tissue section from the PTU pretreated patient. The green in the TOF-SIMS image represents the signal from iodine and the red marks PO_3_^−^ (signal from cell membranes). Iodine signals were located within the follicle lumina, but also within thyrocytes.

**Figure 3:**
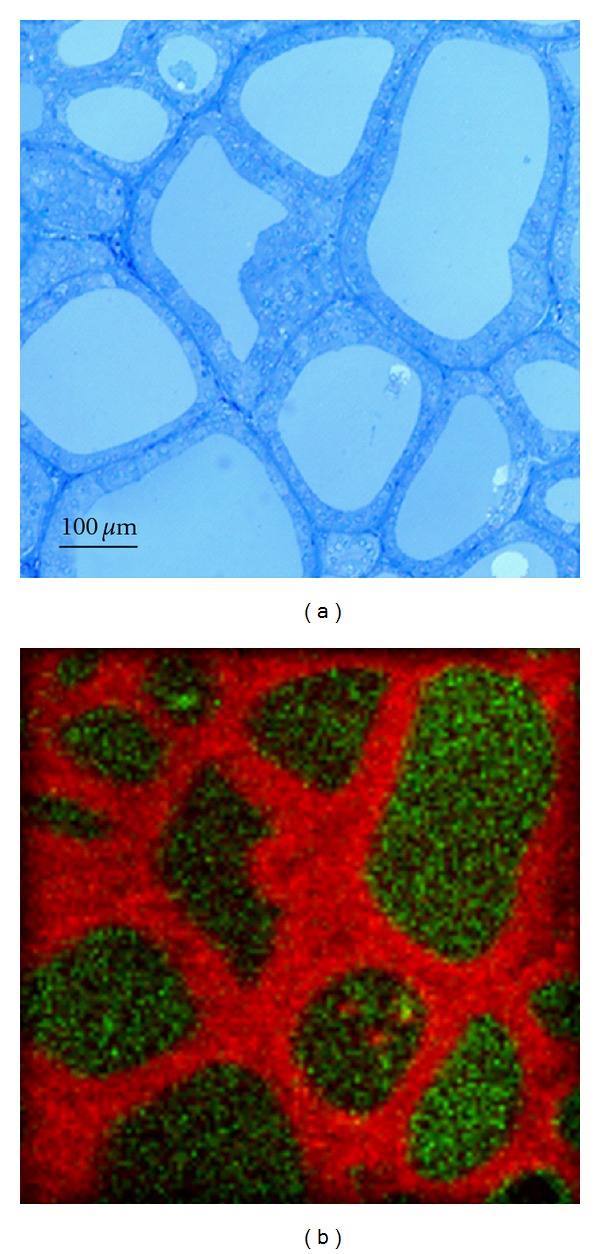
Light microscopy image (a) and TOF-SIMS image (b) of the tissue section from the patient pretreated with stable iodine. The green in the TOF-SIMS image represents the signal from iodine and the red marks PO_3_^−^ (signal from cell membranes). Iodine was located within the follicle lumina. The differences in iodine signal intensity indicate a difference in iodine concentration between the follicle lumina.

 The images in Figures 1, 2, and 3 show typical light micrographs and TOF-SIMS ion images for the samples from the two patients. In both patients, clear signals were detected for iodine. A comparison of the light microscopy slides with the TOF-SIMS images showed that iodine was present within the follicle lumina (Figures 1 and 3). There was variation in iodine concentration between (Figure 3(b)) and within (Figure 1(b)) some follicle lumina. The sample from Patient 1 also showed iodine signals outside the follicle lumina. Magnification of such an area revealed the iodine signals were colocalized with the thyrocytes (Figure 2). In tissue from Patient 2, the patient pretreated with stable iodine, the thyrocytes were enlarged, with a cubical shape corresponding to hyperfunctioning thyrocytes (Figure 3(a)). The thyrocytes in the PTU-treated patient appeared more similar to the cell shape found in subjects with healthy thyroids (Figure 1(a)).

## 3. Discussion

 This pilot study demonstrates the value of the combined TOF-SIMS and XRF results to elucidate the complicated mechanisms of iodine metabolism during hyperthyroidism and its treatment. With TOF-SIMS, it was possible to demonstrate different cellular and extracellular locations of iodine in the two differently treated patients, whereas XRF analysis provided quantitative measures of the iodine concentration. XRF estimates of iodine in surgical tissue have high accuracy [9], and previous studies have shown that iodine loss (predominately as free iodine) due to fixation for TOF-SIMS analysis is small [12]. As to our knowledge, this is the first study using TOF-SIMS and XRF in combination to investigate iodine metabolism in Graves' disease. The analyses, however, are time consuming and thus not applicable for larger studies.

 The patient pretreated with PTU had poor compliance for two years and still showed mild hyperthyroidism at the time of surgery. This patient had a relatively high iodine pool, 0.9 mg/mL, compared to the average thyroid iodine content in healthy individuals in western Sweden, which has been determined to be 0.4 mg/mL [13]. This may be explained by high TSH receptor stimulation by TRAbs and simultaneous incomplete inhibition of TPO-function for a long period of time. Furthermore, the iodine supply in western Sweden is sufficient [14], and it has been shown that patients living under iodine sufficient conditions need ATD for a longer period for successful treatment [15]. The microscopic images of thyrocytes and follicles in this PTU-pre-treated patient resembled the pattern usually seen in normal thyroids. A high iodine concentration could be expected, as the TOF-SIMS images illustrated that the follicle sizes were larger, meaning that there was a relatively larger volume where iodine could be stored. There was large heterogeneity in the iodine signal within some follicle lumina, which is in accordance with the findings of Clerc et al., who also found heterogeneous distribution of iodine stores after ATD treatment [16]. 

 Moreover, using TOF-SIMS analysis, we were able to visualize iodine intracellularly in the thyrocytes as well as in the follicle lumina in the specimen from the PTU-treated patient. The iodine signal seen with TOF-SIMS is interpreted to derive from organically bound iodine rather than from free iodide since free iodide is unlikely to remain at the tissue surfaces. Intracellular iodine findings might partly be due to accumulation of iodinated intermediate compounds engaged at the TPO-complex during iodination of thyroglobulin [2]. The most likely explanation, however, is that the intracellular iodine represents endocytosed iodinated thyroglobulin since PTU does not inhibit this process. Intracellular iodine was earlier not observed in normal thyroid tissue using TOF-SIMS analysis [8]. 

 The second patient showed severe hyperthyroidism before pretreatment with stable iodine ten days before surgery. The light-microscopy images show a cubical shape of the thyrocytes, which is compatible with what is commonly seen in Graves' hyperthyroidism. Larger doses of stable iodine have several effects on the thyroid. The most clinically important is a rapid inhibition of thyroid hormone release from the thyroid cells by inhibition of endocytosis of iodinated thyroglobulin from the follicle lumina, the so-called Plummer effect. This effect is believed to sustain for about two weeks, during which time the surgery has to be performed. Thus, Plummer blockage prevents the iodine store in the follicle lumen from being emptied. Simultaneously with the Plummer effect, a high intracellular iodine concentration is thought to lead to the Wolff-Chaikoff effect, inhibiting iodine organification. The Wolff-Chaikoff effect is reversible (escape), a phenomenon which is attributed to a downregulation of the NIS-protein at the plasma membrane, lowering iodine uptake [17, 18]. After a couple of days, the iodine organification and thyroid hormone production resumes [18]. The iodine concentration, which was comparable to the concentrations found among euthyroid patients, found in this patient with severe hyperthyroidism may have been accumulated before treatment with stable iodine. However, neither the mechanisms governing the Wolff-Chaikoff effect nor the apical iodine transport systems are fully elucidated.

 Earlier investigations of iodine distribution in thyroid tissue, using dynamic SIMS, suggested chronic iodine overload could lead to accumulation of iodine in thyrocytes [7] and in the stroma [6]. Necrosis of thyrocytes has been reported after a high iodine intake [19], which may explain this accumulation. However, stromal iodine was not found in the present study using TOF-SIMS, which has higher mass resolution than dynamic SIMS and thus enables a better histological discrimination.

 Both patients in this paper can be characterised to have a high iodine pool according to the in vivo XRF-studies of Jonckheer et al. [20]. A high iodine pool is considered to be more resistant to therapy which was evident in the present cases. We were not able to perform urine iodine analyses in our patients but this parameter is only an indication of a recent contamination and has earlier not been shown to correlate to the thyroid-iodine-pool [13, 20].

 A considerable thyroid iodine store was detected in specimens from two patients with Graves' disease both after treatment with PTU and stable iodine. The iodine store is deposited extracellular in the follicle lumina, whereas intracellular iodine was only found in the patient treated with PTU, which emphasizes the value of treatment with stable iodine when a prompt stop of hormone release is warranted. A combination of quantitative and qualitative studies using XRF and TOF-SIMS analysis can thus contribute to the study of the intricate processes following the available medical treatment alternatives in Graves' disease.
